# Combined intracoronary nitroprusside infusion during prolonged perfusion balloon inflation for ST-segment elevation myocardial infarction

**DOI:** 10.1093/ehjcr/ytad609

**Published:** 2023-12-01

**Authors:** Hiroyuki Yamamoto, Chiaki Yoshida, Nobuhiro Watanabe, Tomofumi Takaya

**Affiliations:** Division of Cardiovascular Medicine, Department of Internal Medicine, Hyogo Prefectural Harima-Himeji General Medical Center, 3-264 Kamiya-cho, Himeji 670-8560, Japan; Division of Cardiovascular Medicine, Department of Internal Medicine, Hyogo Prefectural Harima-Himeji General Medical Center, 3-264 Kamiya-cho, Himeji 670-8560, Japan; Division of Cardiovascular Medicine, Department of Internal Medicine, Hyogo Prefectural Harima-Himeji General Medical Center, 3-264 Kamiya-cho, Himeji 670-8560, Japan; Division of Cardiovascular Medicine, Department of Internal Medicine, Hyogo Prefectural Harima-Himeji General Medical Center, 3-264 Kamiya-cho, Himeji 670-8560, Japan; Department of Exploratory and Advanced Research in Cardiology, Kobe University Graduate School of Medicine, 7-5-1 Kusunoki-cho, Chuo-ku, Kobe 6500017, Japan

**Keywords:** Intracoronary infusion, Perfusion balloon, Slow-flow phenomenon, Stentless percutaneous coronary intervention, Myocardial infarction, Thrombus

## Case description

**Figure 1:**
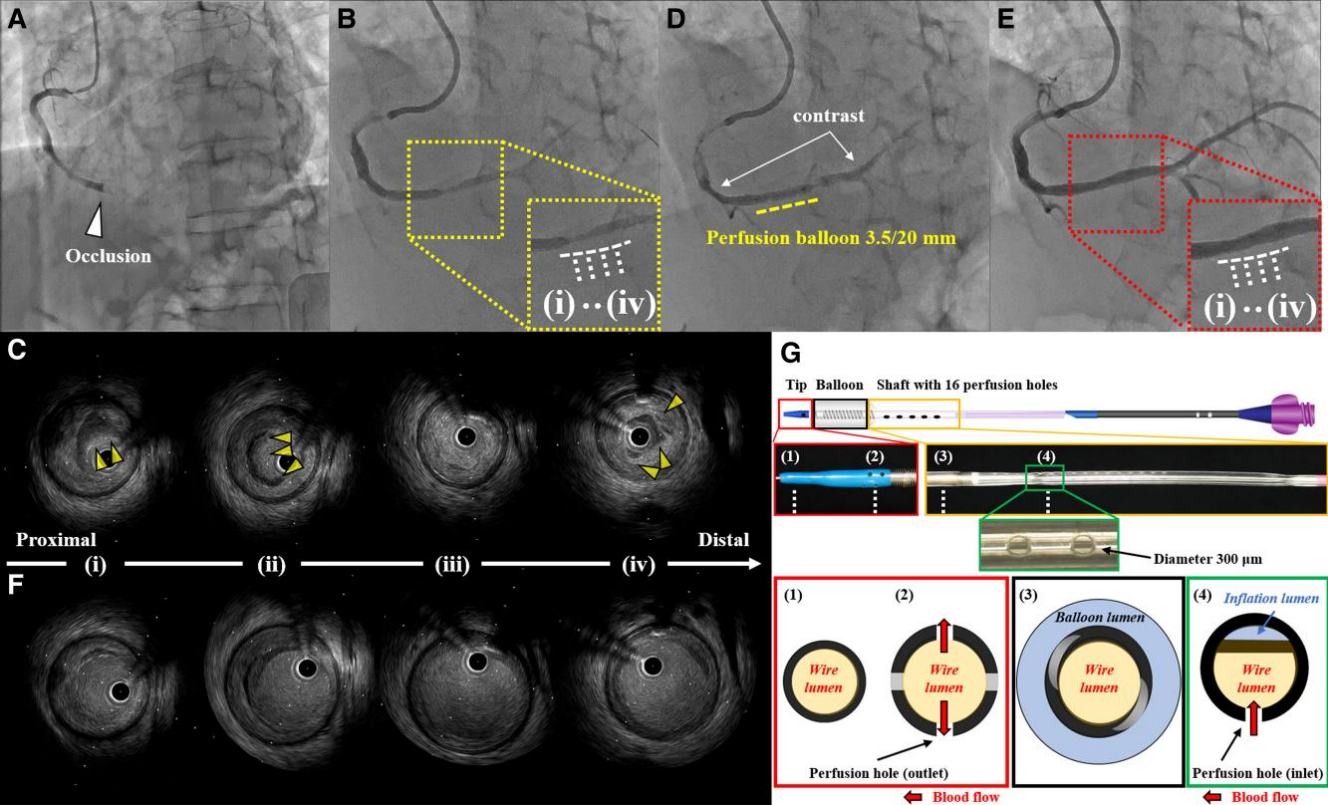
(*A*) Initial and (*B*) post-thromboaspiration coronary angiography. (*C*) Initial intravascular ultrasound showing residual thrombus and plaque in each lesion (i–iv) visible on *B*. (*D*) Post-perfusion balloon angioplasty and final (*E*) coronary angiography. (*F*) Final intravascular ultrasound images showing optimal lumen enlargement. Arrowheads indicate the residual thrombus. (*G*) Structure of a perfusion balloon, which can perfuse blood or intracoronary-infused drugs through wire lumen via multiple side holes in the shaft.

A 51-year-old man who presented with chest pain that had persisted for 19 h was diagnosed with inferior ST-segment elevation myocardial infarction. On admission, the patient’s creatine kinase (CK) and CK-myocardial band (CK-MB) levels were already elevated (3351 U/L and 315 ng/mL). Emergency coronary angiography revealed a mid-right coronary artery occlusion with large thrombi (*Figure 1A*). Repeated thromboaspiration using a 7 Fr Rebirth catheter (Terumo, Tokyo, Japan) eliminated the fresh red thrombus and achieved recanalization (*Figure 1B*). However, intravascular ultrasound (IVUS) confirmed a residual thrombus on the plaque without evident rupture, with persistent coronary flow disturbance (*Figure 1C*). Prolonged balloon inflation using a 3.5/20 mm perfusion balloon (Ryusei, Kaneka, Osaka, Japan) was performed three times for a total of 9 min to compress the thrombus and plaque. In addition, intracoronary sodium nitroprusside infusion (50 µg/dose, three times) was performed during perfusion balloon angioplasty to restore coronary flow, resulting in an angiographically optimal outcome with sufficient coronary flow improvement (*Figure 1D*). Due to the sufficient luminal gain achieved with perfusion balloon angioplasty following thromboaspiration, the subsequent drug-coated balloon angioplasty using a SeQuent Please 4.0/30 mm (B. Braun Melsungen AG, Germany) successfully resulted in stentless PCI (*Figure 1E* and *F*; Supplementary material online, *Videos S1* and *S2*). Subsequently, the highest CK and CK-MB levels were 4867 U/L and 401 ng/mL, respectively. Transthoracic echocardiography showed focal left ventricular asynergy in the inferior myocardium with a reduced left ventricular ejection fraction of 48%. Thereafter, the patient had an uneventful clinical course.

In acute coronary syndrome (ACS) cases, massive coronary thrombus poses clinical challenges, leading to slow/no reflow. Distal embolization caused by balloon angioplasty should be prevented in such situations. Perfusion balloon, which features a central lumen and multiple side holes in the shaft, may allow coronary flow during prolonged inflation and effectively compress the thrombus (*Figure 1G*).^1^ Intracoronary infusion with drugs including adenosine and sodium nitroprusside (available in some regions as strong coronary vasodilators instead of adenosine) provides cardioprotective effects by reducing reperfusion-mediated injury.^2–5^ The presented case demonstrated the utility of combined intracoronary drug infusion during prolonged perfusion balloon inflation, facilitating sufficient luminal gain and coronary flow improvement. This resulted in stentless PCI with drug-coated balloon angioplasty, even in a case of thrombotic ACS. Thus, drug infusion therapy during perfusion balloon angioplasty serves a dual purpose of improving epicardial coronary obstruction and microvascular disorders in the myocardium, increasing the potential for stentless PCI even in challenging ACS cases.

## Supplementary Material

ytad609_Supplementary_Data

## Data Availability

The data underlying this article are available in the article and its online Supplementary material.

## Supplementary material

**Consent:** In line with the COPE guidelines, informed consent was obtained from the patient for their participation in this study and the publication of the case and accompanying images.

**Funding:** None declared.
